# Effectiveness of cervical screening after age 60 years according to screening history: Nationwide cohort study in Sweden

**DOI:** 10.1371/journal.pmed.1002414

**Published:** 2017-10-24

**Authors:** Jiangrong Wang, Bengt Andrae, Karin Sundström, Alexander Ploner, Peter Ström, K. Miriam Elfström, Joakim Dillner, Pär Sparén

**Affiliations:** 1 Department of Medical Epidemiology and Biostatistics, Karolinska Institutet, Stockholm, Sweden; 2 Centre for Research and Development, Uppsala University/Region of Gävleborg, Gävle, Sweden; 3 Department of Laboratory Medicine, Karolinska Institutet, Stockholm, Sweden; 4 Karolinska University Laboratory, Karolinska University Hospital, Stockholm, Sweden; Vanderbilt University School of Medicine, UNITED STATES

## Abstract

**Background:**

The relatively high incidence of cervical cancer in women at older ages is a continuing concern in countries with long-established cervical screening. Controversy remains on when and how to cease screening. Existing population-based studies on the effectiveness of cervical screening at older ages have not considered women’s screening history. We performed a nationwide cohort study to investigate the incidence of cervical cancer after age 60 years and its association with cervical screening at age 61–65, stratified by screening history at age 51–60.

**Methods and findings:**

Using the Total Population Register, we identified 569,132 women born between 1 January 1919 and 31 December 1945, resident in Sweden since age 51. Women’s cytological screening records, cervical cancer occurrence, and FIGO stage (for those diagnosed with cancer) were retrieved from national registers and medical charts. We calculated the cumulative incidence of cervical cancer from age 61 to age 80 using a survival function considering competing risk, and estimated the hazard ratio (HR) of cervical cancer in relation to screening status at age 61–65 from Cox models, adjusted for birth cohort and level of education, conditioning on women’s screening history in their 50s. In women unscreened in their 50s, the cumulative incidence up to age 80 was 5.0 per 1,000 women, and screening at age 61–65 was associated with a lower risk for cervical cancer (HR = 0.42, 95% CI 0.24–0.72), corresponding to a decrease of 3.3 cancer cases per 1,000 women. A higher cumulative incidence and similarly statistically significant risk decrease was seen for women with abnormal smears in their 50s. In women adequately or inadequately screened with only normal results between age 51 and age 60, the cumulative incidence of cervical cancer from age 61 to 80 was 1.6 and 2.5 per 1,000 women, respectively, and further screening at age 61–65 was not associated with statistically significant decreases of cervical cancer risk up to age 80, but with fewer cancer cases of advanced stages at age 61–65. Adjustment for potential lifestyle confounders was limited.

**Conclusions:**

In this study, cervical screening with cytology at age 61–65 was associated with a statistically significant reduction of subsequent cervical cancer risk for women who were unscreened, or screened with abnormalities, in their 50s. In women screened with normal results in their 50s, the risk for future cancer was not sizeable, and the risk reduction associated with continued screening appeared limited. These findings should inform the current debate regarding age and criteria to discontinue cervical screening.

## Abstract 2

Jiangrong Wang and colleagues show in their cytology screening cohort that women who test negative for cervical cancer in their 50s are at minimal risk for developing cancer in their 60s.

## Author summary

**Why was this study done?:**

- Cervical cancer in women aged 30–60 years has been significantly reduced in countries performing cervical screening, whereas cancer among older women has not been reduced to the same extent.
- Controversy remains on whether and how to perform cervical screening among older women, in order to prevent cancer at older ages.
- Previous studies have shown only the pooled effect of cervical screening in women aged 50 and above.
- Detailed evidence is lacking about at which age and with which criteria to discontinue cervical screening; previous screening history may be an important factor determining cancer risk and the impact of further screening among older women.

**What did the researchers do and find?:**

- We performed a nationwide cohort study in more than half a million women in Sweden, using Swedish national registry data on cervical screening, cancer, education, death, migration, and hysterectomy. Cytological screening was in use over the study period.
- The risk of cervical cancer after age 60 by screening history at age 51–60 was assessed, and the risk decrease associated with screening at age 61–65 was compared among women with different screening histories in their 50s.
- We found that women who were unscreened, or screened with abnormal results, in their 50s had a relatively high risk of cervical cancer after age 60, and being screened at age 61–65 was associated with an evident risk decrease up to age 80. In women who were screened in their 50s and had only normal results, both cancer risk and the risk decrease associated with screening after age 60 were not sizable.

**What do these findings mean?:**

- The impact of cervical screening after age 60 on the risk of cervical cancer up to age 80 varied depending on women’s screening history at age 51–60.
- Screening strategies for women above age 60 could be differential depending on earlier screening history.

## Introduction

The relatively higher incidence of cervical cancer in women older than 60 years as compared to women at age 45–60 years has become a concern in countries with long-established cervical screening [1,2]. The older aged cases still account for more than one-third of the annual cervical cancer case load, and are also found at more advanced stages [3,4]. Cervical screening, which has been mainly provided to women up to age 50–60 [5–8], is being extended to the age of 60–65 or 70 in countries facing high incidence of cervical cancer in older women [5,9,10]. However, the age at which to cease cervical screening, and with which criteria, remains controversial, as the existing evidence is insufficient.

A comprehensive investigation of cervical cancer incidence and the effectiveness of cervical screening at older ages is crucial for assessing when and how to cease cervical screening. Although the incidence in older women is higher than among middle-aged women, this difference may not represent the biological age-specific risk profile, since the 2 age strata at the same calendar time represent different birth cohorts with different screening backgrounds. A recent ecological study based on data from 4 Nordic countries reported that the high incidence after age 60 was not observed among the younger birth cohorts, which were more likely to have satisfactory screening history [11]. This underscores the importance of screening history in predicting cervical cancer development at older ages, and hints at the possibility that the effectiveness of cervical screening in older women may also diverge depending on screening history. This was suggested in studies reporting fewer precursor lesions after reproductive ages following previous negative screening results [12,13]. Yet, no population-based study assessing the effectiveness of cervical screening at older ages with the endpoint measure of invasive cervical cancer has considered previous screening history [4,9,14,15].

There are currently decades of data gathered from screening with cytology in the Swedish population that could serve to inform this issue. The Swedish context—with a national cervical screening registry, electronic registration of the entire population, and comprehensive cancer registration since the 1960s—provides a unique opportunity to investigate the incidence of cervical cancer after age 60 and its association with cervical screening at age 61–65, stratified by women’s screening history in their 50s.

## Methods

### Ethical permit

The Regional Ethics Committee in Stockholm, Sweden, granted ethical approval for this study and concluded that informed consent from the women was not required.

### Cervical screening for older aged women and the cervical screening registry in Sweden

Sweden has had an organized cervical screening program covering the whole population since the early 1970s. Initially, the upper limit for invitation was age 50, which was extended to age 60 in the 1998 national guidelines [7]. Due to variations in implementation, some regional programs have been offering screening up to age 65. Papanicolaou (Pap) test was the tool of screening over the study period, and primary HPV screening did not start for women aged above 50 before the end of the study period.

The Swedish National Cervical Screening Registry (NKCx) collects records of all organized and opportunistic cervical screening tests in Sweden [16]. Computerized registration began successively by county in 1970, with complete nationwide coverage of the NKCx from 1995 onwards (S1 Fig).

### Study population and data source

**Fig 1:**
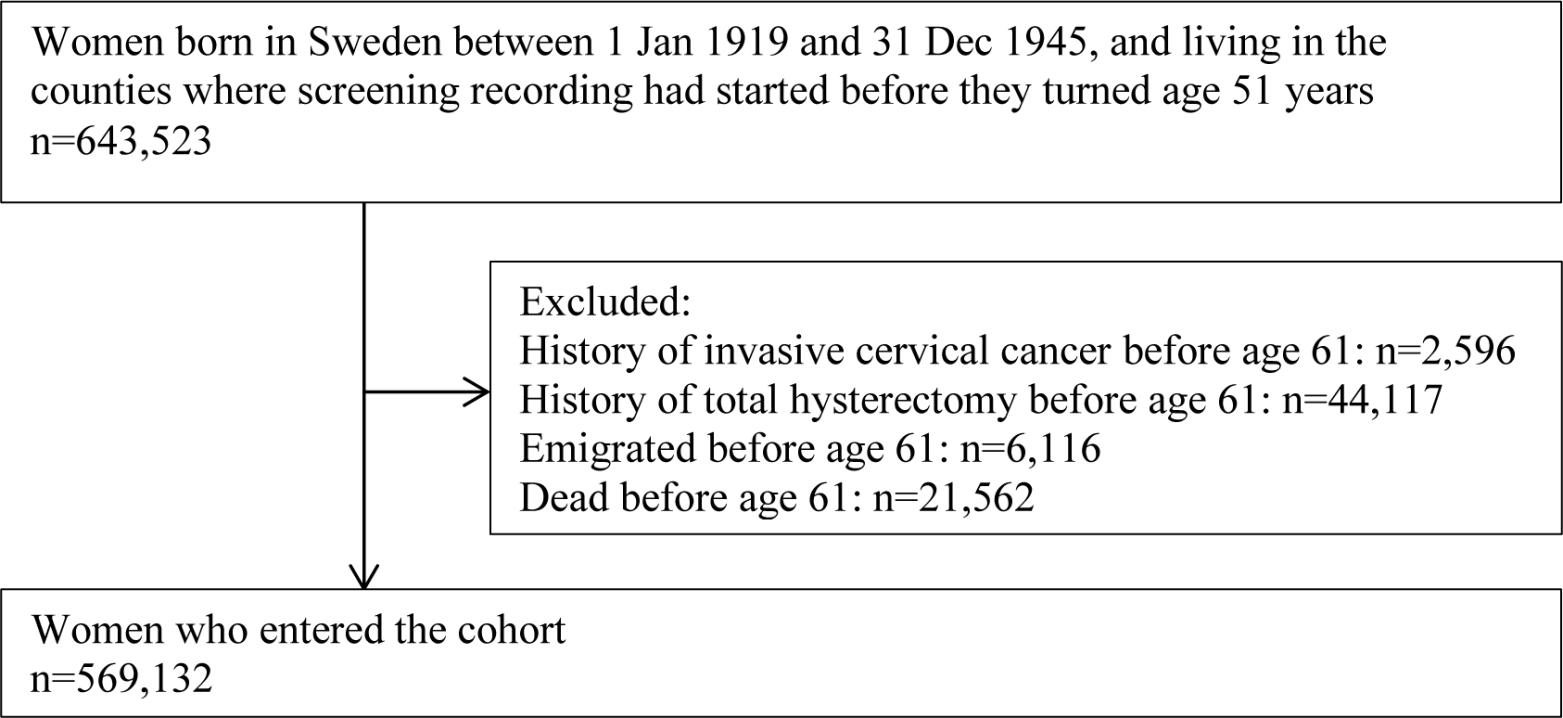
Flow chart for the study population.

This nationwide cohort study included women born between 1 January 1919 and 31 December 1945, i.e., 51 years of age or younger in 1970 and 66 years or older by the end of 2011, resident in Sweden since age 51, and at age 51 or younger when their county of residence started to record cervical screening (Fig 1).

Women who died or emigrated before age 61 years or who had invasive cervical cancer or total hysterectomy before age 61 years were excluded (Fig 1) as they were not at risk for a first diagnosis of cervical cancer at age 61 years or above. The remaining women entered the cohort when they turned 61 years of age, and were followed until a diagnosis of invasive cervical cancer, a total hysterectomy, emigration from Sweden, age 81, death, or 31 December 2011, whichever came first.

We used the Swedish Total Population Register (TPR) to identify the study population [17], used NKCx to retrieve information on the date and result of Pap tests, and used the Swedish National Cancer Registry (NCR) to identify diagnoses of invasive cervical cancer [18]. The NCR contains complete records of all cancer diagnoses in Sweden since 1958. Information on education level, hysterectomy, emigration, and death was collected from the National Education Register in the Longitudinal Integration Database for Health Insurance and Labour Market Studies [19], the National Patient Register [20], TPR, and the Swedish Causes of Death Register [21], respectively. All data were linked via the unique Swedish personal identification number and then anonymized by Statistics Sweden [22].

### Screening status

The screening history of women at age 51–60 was categorized as follows: (1) “adequately screened with normal results,” if women had (a) at least 1 Pap test with normal result at age 51–55 and 1 Pap test with normal result at age 56–60, (b) the first and last test at least 1 year apart, and (c) no abnormal result at age 51–60; (2) “inadequately screened with normal results,” if women had 1 or more Pap tests with only normal results, but only in 1 age span (51–55 or 56–60 years); (3) “unscreened,” if women had no Pap test at age 51–60; (4) “low-grade abnormality,” if women had ASCUS (atypical squamous cells of undetermined significance) or mild squamous dysplasia at least once at age 51–60, but not any high-grade abnormality; (5) “high-grade abnormality,” if women had moderate or severe squamous dysplasia, atypical glandular cells [23], atypical cells of uncertain origin, adenocarcinoma in situ, cytological implication of squamous cell cancer, adenocarcinoma, or malignancy of uncertain origin at least once at age 51–60. SNOMED codes were used to define the Pap test results (Table A in S2 Text).

We identified each woman’s first Pap test record at age 61–65 as the main exposure of interest. Pap tests within 50 days prior to cervical cancer diagnosis were disregarded, to exclude tests that formed part of the diagnostic workup. The 50-day time frame was verified among cervical cancer cases during 2002–2011, where information on mode of detection (i.e., screen-detected or symptomatic cancer) was collected from medical charts (S2 Fig). Subsequently, screening status at age 61–65 was treated as a time-varying covariate, meaning that women contributed “unscreened” risk time before the date of the first screening Pap test, and contributed “screened” risk time thereafter.

### Cervical cancer occurrence

We used the International Classification of Diseases–7th Revision (ICD-7) code 171 to identify the occurrence and date of invasive cervical cancer for each study participant. The FIGO stage (International Federation of Gynecology and Obstetrics staging system) of cervical cancer cases in 2002–2011 was collected from medical charts, and reviewed by a senior gynecologist (BA) and senior pathologist (Walter Ryd). Microinvasive (stage IA), localized (stage IB), and advanced (stage II+) cancer were classified for examining the effect of screening on reducing advanced cervical cancer (downstaging).

### Confounding factors

Education is a potential confounder for the association between screening status and cervical cancer development since it can be a marker of both health consciousness and lifestyle. We identified the highest education level of each woman before age 51 years, categorized as (1) low (less than high school), (2) middle (high school), (3) high (university exam and above), or (4) missing, and included this variable in the analyses.

Birth cohort is also an important potential confounder, as it may reflect an underlying difference in risk of cervical cancer between generations, as well as screening status at age 61–65, due to the age extension of invitations to screening with calendar time. Birth cohort was categorized into 5 groups: 1919–1925, 1926–1930, 1931–1935, 1936–1940, and 1941–1945.

### Statistical analyses

To assess the cancer preventive effect of cervical screening at age 61–65, we modeled our data in a competing risk framework [24], with cervical cancer as the primary outcome and total hysterectomy and death as competing events. We present crude event probabilities as cumulative incidence curves of cervical cancer at age 61–80 by screening status at age 61–65, stratified by screening history at age 51–60. We quantified the effect of screening status at age 61–65 on risk of cervical cancer by calculating the absolute risk difference of cumulative incidence, as well as the relative risk using both cause-specific hazard ratios (HRs) based on a standard Cox model and sub-distribution HRs based on a Fine–Gray model for the cumulative incidence function [25]. Both types of HRs were calculated for all 5 classes of screening history at age 51–60 separately, both unadjusted and adjusted for level of education and birth cohort.

For the adjusted models, we performed a number of sensitivity analyses to explore possible effects of underlying assumptions. (1) The assumption that there was no confounding by smoking and parity was explored by including a diagnosis of chronic obstructive pulmonary disease (COPD) as a proxy for smoking and number of children, retrieved from the National Patient Register [20] and the Multi-Generation Register [26], respectively. (2) The assumption that there was no differential effect due to varied screening coverage between counties was explored by limiting the analysis to counties with more than 40% screening coverage between age 61 and age 65. (3) The assumption that there was no differential effect due to screening registry availability and period effect was explored by restricting the analysis to later birth cohorts (1926–1945, 1931–1945, and 1936–1945). (4) The robustness of the distinction between screening and diagnostic Pap tests was explored by varying the cutoff time separating the two. (5) To assess the potential differences between women included in and excluded from the study population due to the completeness of screening registry, we compared the level of education of the 2 groups adjusted for birth cohort.

For cervical cancer cases diagnosed in 2002–2011, FIGO stage at diagnosis was tabulated as a function of screening status at age 61–65, stratifying by screening history at age 51–60. The relationship between stage and screening status was modeled via a proportional odds ratio model with a cumulative logit link [27]. The proportional odds ratio is a measure of the risk of being diagnosed with later stage disease in unscreened women.

Data management was performed with SAS statistical package version 9.4. Statistical analyses were conducted in STATA version 14.

There is no registered or published analysis protocol for this study. We present the original and revised analysis plan with reasons for changes in S1 Text.

## Results

### Study population

We identified 643,523 women born between 1 January 1919 and 31 December 1945 who were resident in Sweden since age 51 and who were age 51 or younger when their county of residence initiated registration of cervical screening. The difference in level of education among women included in and excluded from the study population due to screening record availability was statistically significant (due to sample size) but minimal, after adjustment for birth cohort (Table B in S2 Text). Between the ages of 51 and 60 years, 2,596 women were diagnosed with invasive cervical cancer, 44,117 had a total hysterectomy, and 27,678 died or emigrated. The remaining 569,132 women entered the study cohort (Fig 1).

### Characteristics of the study cohort

In the study cohort, 63% of women were not screened at age 61–65 years, while 37% were. The screened group had a higher level of education (Table 1). A majority of women (60%) in the study cohort were adequately screened with normal results in their 50s (age 51–60). Women screened at age 61–65 were adequately screened to a higher extent (72%) and unscreened to a lower extent (5%) in their 50s, compared to those unscreened at age 61–65 (52% and 22%, respectively) (Table 1). There were in total 868 cervical cancer cases diagnosed at age 61–80 in the study population. The majority of cases were in women unscreened at age 61–65 (76%), and these cases also presented at more advanced FIGO stages than cases screened during this age span (Table 1).

**Table 1:** Characteristics of the study population, by cervical screening status at age 61–65 years.

Characteristic	Unscreened	Screened	Total
**Number of women**	360,093 (63)	209,039 (37)	569,132 (100)
**Mean follow-up time (years)**	10.6	11.4	10.9
**Birth cohort**			
1919–1925	18,500 (5)	8,272 (4)	26,772 (5)
1926–1930	33,886 (10)	14,846 (7)	48,723 (9)
1931–1935	51,765 (14)	40,707 (19)	92,472 (16)
1936–1940	85,662 (24)	62,664 (30)	148,326 (26)
1941–1945	170,280 (47)	82,550 (40)	252,830 (44)
**Education**			
Low	153,489 (42)	76,105 (37)	229,594 (40)
Middle	135,919 (38)	81,732 (39)	217,651 (38)
High	67,802 (19)	50,660 (24)	118,462 (21)
Missing	2,883 (1)	542 (0)	3,425 (1)
**Screening history at age 51–60**			
Adequately screened, normal	189,287 (52)	151,416 (72)	340,703 (60)
Inadequately screened, normal	82,139 (23)	29,727 (14)	111,866 (19)
Unscreened	78,665 (22)	10,507 (5)	89,172 (16)
Low-grade abnormality	6,047 (2)	9,894 (5)	15,941 (3)
High-grade abnormality	3,955 (1)	7,495 (4)	11,450 (2)
**Number of cervical cancer cases at age 61–80**	662 (0.184)	206 (0.098)	868 (0.152)
**FIGO stage of cervical cancer cases**			
IA (microinvasive)	13 (2)	17 (8)	30 (4)
IB (localized)	122 (18)	53 (26)	175 (20)
II+ (advanced)	249 (38)	65 (32)	314 (36)
Missing^a^	278 (42)	71 (34)	349 (40)

Data are given as number (percent) unless otherwise indicated.

^a^FIGO stage information was only available for cervical cancer cases from 2002–2011; thus, the stage of earlier cases was missing.

### Risk of cervical cancer at age 61–80 years

**Fig 2:**
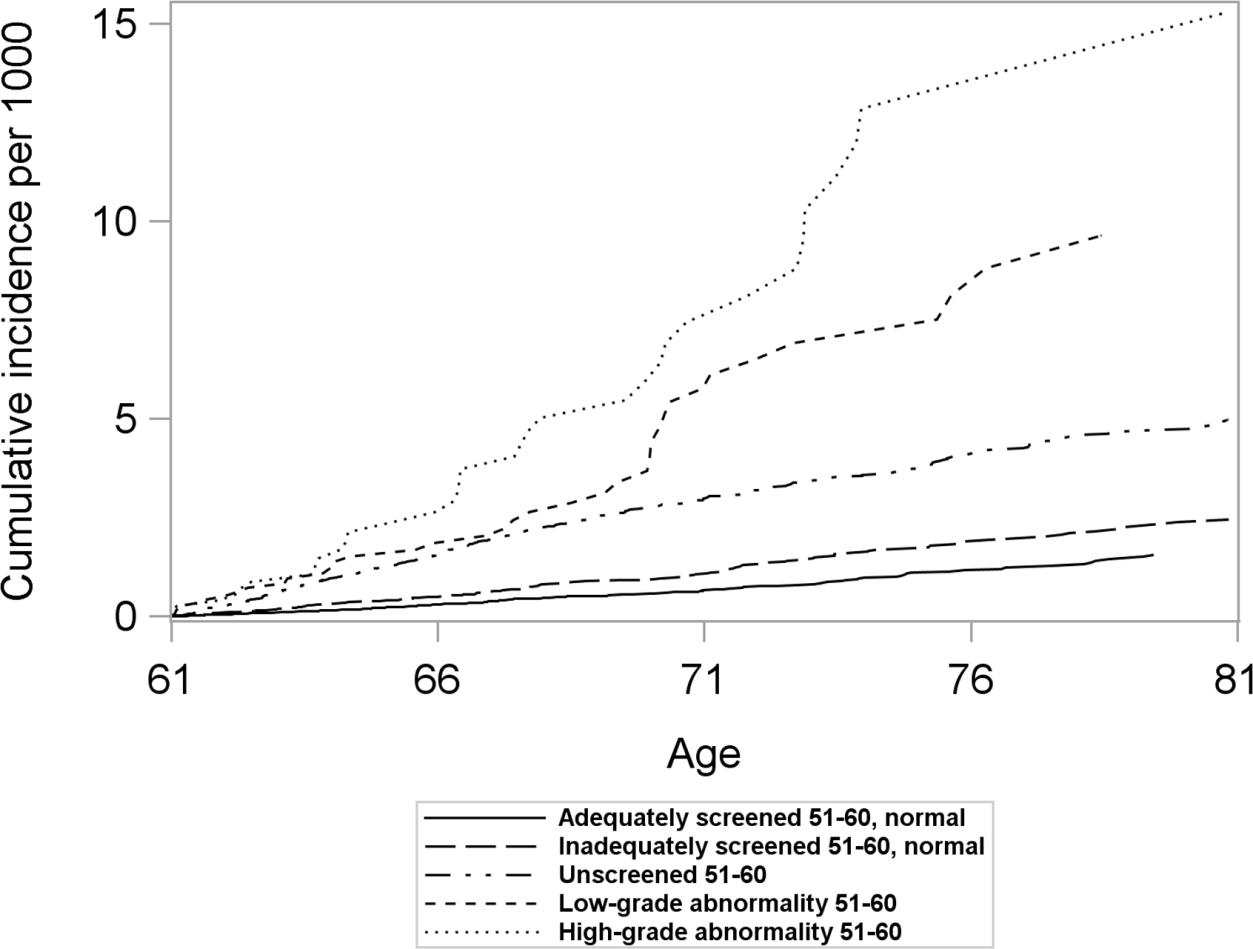
Cumulative incidence of cervical cancer from age 61 to 80 by screening history at age 51–60 in women unscreened after age 60, considering death and total hysterectomy as competing events. Women screened after age 60 were censored at the time of their first Pap test after age 60.

**Fig 3:**
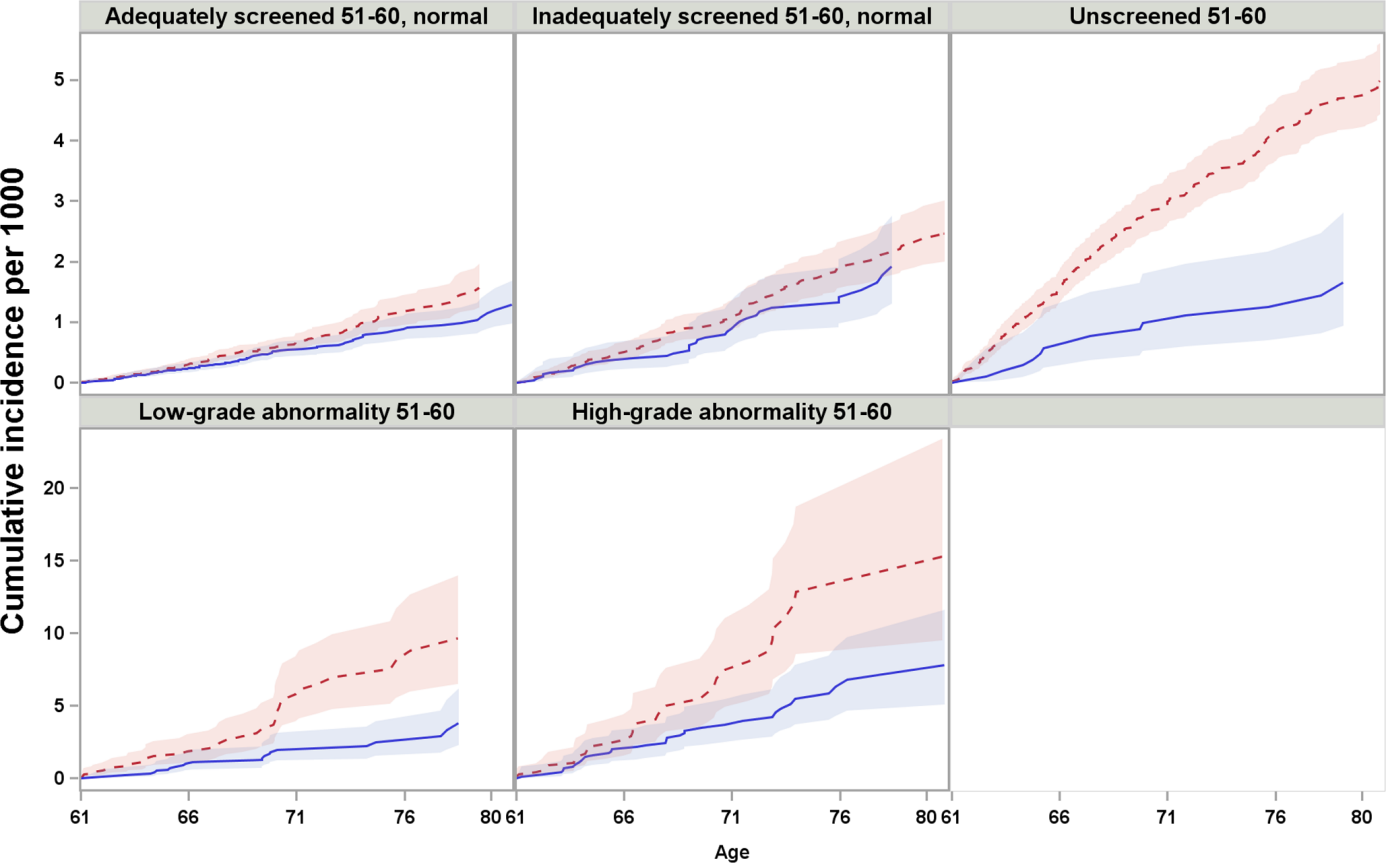
Cumulative incidence and 95% confidence intervals of cervical cancer among women screened and unscreened at age 61–65, by screening history at age 51–60, considering death and total hysterectomy as competing events. Red dotted line: unscreened at age 61–65; blue solid line: screened at age 61–65. Note that the scales of *y*-axis are different between the first and second row.

Women with differing screening histories in their 50s exhibited different risk profiles for cervical cancer (Fig 2). The cumulative incidence of cervical cancer from age 61 to age 80 was highest for women with high-grade abnormalities in their 50s and lowest for women adequately screened with normal results (Fig 2). Across all screening history groups, women screened at age 61–65 exhibited a lower cumulative incidence of cervical cancer between age 61 and 80 than those who were not screened (Fig 3). However, the size of the difference varied depending on screening history (Fig 3).

For women who were adequately or inadequately screened with normal results in their 50s, and then were unscreened after age 60, the cumulative incidence of cervical cancer up to age 80 was 1.6 and 2.5 per 1,000 women, respectively. Screening at age 61–65 yielded an estimated decrease of 0.3 and 0.5 cases per 1,000 women (Table 2) in previously adequately and inadequately screened women, respectively. These decreases corresponded to 10% (HR = 0.90, 95% CI 0.69–1.17) and 18% (HR = 0.82, 95% CI 0.56–1.22) lower hazards, respectively, which were not statistically significant (Table 3).

**Table 2:** Number of cervical cancer cases, cumulative incidence, and cumulative incidence difference among women screened and unscreened at age 61–65, by screening history at age 51–60, for every 5 years of follow-up from age 61 to age 80, considering death and total hysterectomy as competing events.

Screening history at age 51–60	Screening status at age 61–65	By age 65			By age 70			By age 75			By age 80		
		Cum *N*^a^	Cum. inc. per 1,000 (95% CI)^b^	Cum. inc. difference per 1,000 (95% CI)^c^	Cum *N*^a^	Cum. inc. per 1,000 (95% CI)^b^	Cum. inc. difference per 1,000 (95% CI)^c^	Cum *N*^a^	Cum. inc. per 1,000 (95% CI)^b^	Cum. inc. difference per 1,000 (95% CI)^c^	Cum *N*^a^	Cum. inc. per 1,000 (95% CI)^b^	Cum. inc. difference per 1,000 (95% CI)^c^
Adequately screened, normal	Unscreened	73	0.31 (0.24–0.39)		119	0.66 (0.55–0.80)		144	1.17 (0.95–1.44)		153	1.57 (1.24–1.96)	
	Screened	36	0.24 (0.17–0.33)	−0.07 (−0.17, 0.04)	72	0.54 (0.43–0.69)	−0.12 (−0.30, 0.06)	92	0.88 (0.70–1.10)	−0.29 (−0.60, 0.02)	100	1.28 (0.97–1.68)	−0.28 (−0.78, 0.22)
Inadequately screened, normal	Unscreened	47	0.51 (0.38–0.67)		83	1.07 (0.86–1.33)		114	1.90 (1.55–2.32)		125	2.46 (1.99–3.01)	
	Screened	11	0.37 (0.20–0.65)	−0.14 (−0.40, 0.13)	23	0.90 (0.59–1.35)	−0.17 (−0.61, 0.27)	31	1.42 (0.97–2.03)	−0.48 (−1.13, 0.16)	35	1.92 (1.30–2.76)	−0.54 (−1.42, 0.34)
Unscreened	Unscreened	129	1.56 (1.31–1.85)		226	2.99 (2.62–3.40)		280	4.11 (3.64–4.63)		309	4.99 (4.42–5.60)	
	Screened	6	0.57 (0.25–1.22)	−0.98 (−1.52, −0.45)	10	0.99 (0.52–1.79)	−2.00 (−2.73, −1.27)	12	1.25 (0.69–2.16)	−2.86 (−3.73, −1.99)	14	1.66 (0.93–2.80)	−3.33 (−4.41, −2.24)
Low-grade abnormality	Unscreened	17	1.85 (1.10–2.99)		32	5.77 (3.88–8.35)		37	8.15 (5.51–11.70)		39	9.65 (6.46–13.96)	
	Screened	10	1.01 (0.53–1.83)	−0.84 (−1.95, 0.28)	17	1.97 (1.19–3.13)	−3.81 (−6.21, −1.40)	19	2.47 (1.51–3.91)	−5.67 (−8.96, −2.39)	22	3.81 (2.25–6.16)	−5.84 (−10.02, −1.66)
High-grade abnormality	Unscreened	15	2.64 (1.52–4.38)		28	7.46 (4.88–11.02)		35	12.87 (8.55–18.70)		36	15.26 (9.49–23.38)	
	Screened	15	2.00 (1.18–3.26)	−0.64 (−2.36, 1.09)	25	3.70 (2.45–5.42)	−3.76 (−7.13, −0.39)	33	6.31 (4.29–9.03)	−6.56 (−12.11, −1.01)	35	7.81 (5.08–11.59)	−7.46 (−15.04, 0.13)

^a^Cumulative number of cervical cancer cases.

^b^Cumulative incidence and 95% confidence interval, per 1,000 women.

^c^Cumulative incidence difference and 95% confidence interval, per 1,000 women.

**Table 3:** Cause-specific hazard ratio of cervical cancer from age 61 to age 80 comparing women screened and unscreened at age 61–65, by screening history at age 51–60, based on Cox regression model.

Screening history at age 51–60	Unadjusted model			Adjusted for birth cohort and education		
	HR (95% CI)^a^	*P* value	*P* value for PH test^b^	HR (95% CI)^a^	*P* value	*P* value for PH test^b^
Adequately screened, normal	0.92 (0.71–1.20)	0.53	0.27	0.90 (0.69–1.17)	0.42	0.34
Inadequately screened, normal	0.87 (0.60–1.28)	0.49	0.48	0.82 (0.56–1.22)	0.33	0.33
Unscreened	0.38 (0.22–0.66)	<0.01	0.62	0.42 (0.24–0.72)	<0.01	0.28
Low-grade abnormality	0.40 (0.24–0.70)	<0.01	0.41	0.43 (0.25–0.74)	<0.01	0.59
High-grade abnormality	0.58 (0.36–0.94)	0.03	0.23	0.59 (0.36–0.96)	0.04	0.15

^a^Hazard ratio and 95% confidence interval of cervical cancer among women screened at age 61–65 in relation to women unscreened at age 61–65.

^b^Proportional hazard testing based on Schoenfeld residual test.

For women unscreened in their 50s, the cumulative incidence of cervical cancer up to age 80 was 5.0 per 1,000 women when unscreened since age 61, and screening at age 61–65 yielded an decrease of 3.3 cases per 1,000 women (Table 2), corresponding to a 58% (HR = 0.42, 95% CI 0.24–0.72) lower hazard, which was statistically significant (Table 3). In the group of women screened at age 61–65, the cumulative incidence was comparable to those who were screened with normal results in their 50s (Table 2).

For women with low- or high-grade abnormalities in their 50s, the cumulative incidence of cervical cancer up to age 80 was 9.7 and 15.3 per 1,000 women, respectively, when unscreened since age 61. Screening at age 61–65 was associated with estimated decreases of 5.8 and 7.5 cases per 1,000 women (Table 2), corresponding to 57% (HR = 0.43, 95% CI 0.25–0.74) and 41% (HR = 0.59, 95% CI 0.36–0.96) hazard reductions, respectively, which were statistically significant (Table 3).

The sub-distribution HRs from the Fine–Gray model were very similar to the HRs from the Cox model, indicating that the cause-specific HRs in Table 3 capture the differences in cumulative incidence adequately (Table C in S2 Text). The sensitivity analyses limited to counties where more than 40% of women aged 61–65 were screened, adjusting for COPD and parity, and with different time frames for distinguishing screening and diagnostic tests showed similar HRs as the main analysis (Table D in S2 Text). The sensitivity analyses restricting to later birth cohorts also displayed HRs comparable to the main analysis (Table E in S2 Text).

### FIGO stage distribution

Among all cervical cancer cases, women screened at age 61–65 were more likely to be diagnosed with microinvasive or localized cervical cancer at age 61–65 than those unscreened—a finding that was noted in almost all screening history groups. This difference was statistically significant in women adequately screened with normal results, as well as in those unscreened in their 50s. Screening status at age 61–65 was not associated with the proportion of advanced cervical cancer cases after age 65 (Table 4).

**Table 4:** Stage distribution of cervical cancer at age 61–80 (diagnosed in 2002–2011) among women screened and unscreened at age 61–65, by screening history at age 51–60.

Screening history at age 51–60	Screening status at age 61–65	Cervical cancer at age 61–65 years						Cervical cancer at age 66–80 years					
		IA	IB	II+	Total	PropOR (95% CI)^a^	*P* value^b^	IA	IB	II+	Total	PropOR (95% CI)	*P* value^b^
Adequately screened, normal	Unscreened	2 (4.4)	24 (53.3)	19 (42.2)	45 (100.0)	Ref.		5 (8.5)	25 (42.4)	29 (49.2)	59 (100.0)	Ref.	
	Screened	4 (21.0)	11 (57.9)	4 (21.0)	19 (100.0)	0.3 (0.1 to 0.9)	0.03	6 (12.8)	20 (42.5)	21 (44.7)	47 (100.0)	0.8 (0.4 to 1.7)	0.54
Inadequately screened, normal	Unscreened	0 (0.0)	10 (47.6)	11 (52.4)	21 (100.0)	Ref.		0 (0.0)	15 (30.6)	34 (49.4)	49 (100.0)	Ref.	
	Screened	1 (14.3)	3 (42.9)	3 (42.9)	7 (100.0)	0.5 (0.1 to 2.8)	0.44	2 (11.8)	5 (29.4)	10 (58.8)	17 (100.0)	0.5 (0.2 to 1.6)	0.27
Unscreened	Unscreened	1 (1.6)	9 (14.8)	51 (83.6)	61 (100.0)	Ref.		2 (1.9)	28 (26.2)	77 (72.0)	107 (100.0)	Ref.	
	Screened	1 (20.0)	2 (40.0)	2 (40.0)	5 (100.0)	0.1 (0.0 to 0.7)	0.02	1 (12.5)	1 (12.5)	6 (75.0)	8 (100.0)	1.0 (0.2 to 4.9)	1.00
Low-grade abnormality	Unscreened	1 (14.3)	3 (42.9)	3 (42.9)	7 (100.0)	Ref.		0 (0.0)	2 (18.2)	9 (81.8)	11 (100.0)	Ref.	
	Screened	0 (0.0)	2 (33.3)	4 (66.7)	6 (100.0)	3.1 (0.3 to 28.9)	0.32	1 (12.5)	2 (25.0)	5 (62.5)	8 (100.0)	0.3 (0.0 to 2.7)	0.30
High-grade abnormality	Unscreened	1 (11.1)	3 (33.3)	5 (55.6)	9 (100.0)	Ref.		1 (6.7)	3 (20.0)	11 (73.3)	15 (100.0)	Ref.	
	Screened	0 (0.0)	6 (75.0)	2 (25.0)	8 (100.0)	0.4 (0.1 to 2.8)	0.38	1 (10.0)	1 (10.0)	8 (80.0)	10 (100.0)	1.4 (0.2 to 9.0)	0.76

Values for cervical cancer stage are number (percent).

^a^Proportional odds ratio (PropOR) and 95% confidence interval from logistic regression, measuring the relative risk of having a higher stage cancer for screened women as compared to unscreened women.

^b^From Wald test.

## Discussion

### Main findings and interpretations

We showed that women with differing cytological screening histories in their 50s displayed large variability in risk of cervical cancer after age 60. Although all women appeared to benefit from screening at age 61–65, the extent of benefit diverged depending on their screening history in their 50s. Women with a low- or high-grade abnormality in their 50s had the highest risk of cervical cancer after age 60, and further screening was significantly associated with a reduced cervical cancer incidence. Women unscreened in their 50s also faced a non-negligible cancer risk, and further screening was associated with a substantially reduced incidence of cervical cancer up to age 80, and also with significant downstaging of cervical cancer at age 61–65. For women who were adequately or inadequately screened with only normal results in their 50s, the subsequent risk of cervical cancer was much lower than for other groups, and the estimated reduction in cervical cancer incidence from further screening was much smaller, and not statistically significant. However, continued screening in these groups was associated with downstaging of cervical cancers found at age 61–65, especially for women who were previously adequately screened with normal results.

Our findings are in line with the present understanding of the natural history of cervical cancer development as well as the rationale of cervical screening. Women unscreened or screened with abnormalities in their 50s are at higher risk of developing cervical cancer due to the existence of precursor lesions, and later screening may detect and treat advanced lesions, thus significantly decreasing the subsequent risk. In women screened with normal results in their 50s, cervical cancer at older ages may be less likely to develop, considering the generally less intense exposure to HPV after reproductive age. This is in consonance with previous findings that there were fewer precursor lesions after reproductive age following normal screening results [12,13]. Continued screening after age 60 is therefore less likely to be effective due to the lower prevalence of precursors. We found, in supplementary analysis, that the cancer risk decrease associated with screening at age 56–60 tended to be greater than the decrease associated with screening at age 61–65 for those who previously tested normal (although this difference was not statistically significant) (Table F in S2 Text). Despite this, women screened with normal results during their 50s could still benefit from further screening due to downstaging of cancer cases. Lower FIGO stage of cervical cancer at diagnosis is strongly associated with cervical cancer cure, as more than 90% of screening-detected stage IA or IB cancers can be cured [28].

### Comparisons with other studies

Several studies using a case–control design have indicated a benefit of screening at older ages. A US study reported a 77% risk reduction in women aged 55–79 due to screening [14]. A British study found that cervical cancer at age 55–69 could be reduced by 73% with previous 5-yearly screening [15]. Another British study showed that women who were adequately screened at age 50–64 had an 84% risk reduction compared to a unscreened group, up to age 83 [9]. A Finnish study assessed the effectiveness of screening in the same age span as ours, and found that women attending organized screening between the ages of 60 and 64 had a reduced cancer risk of 51% in the following 5 years [4]. This is close to our findings among women unscreened in their 50s. To the best of our knowledge, however, our study is the first population-based cohort study to provide comprehensive evidence about the long-term benefit of screening at age 61–65 in terms of the varying benefit to women with differing screening histories. In the past, older cohorts generally had lower lifetime screening participation; thus, studies grouping all women together irrespective of screening history tended to show a highly significant effectiveness. Today, many women at age 61–65 have been adequately screened, and given our results, the cost-efficiency of screening at these ages may need to be reconsidered. Our study provides detailed, up-to-date evidence for populations with different screening histories, for screening of women over the age of 60.

### Strengths and limitations

We used high-quality national databases and regional variation in the implementation of national cervical screening guidelines to assess cervical screening, cervical cancer, and other health-related and demographic outcomes according to screening history. Our ability to perform individual linkages on hysterectomy, emigration, death, and educational level as well as data on Pap tests taken in both organized and opportunistic screenings enhances the validity and potential for causal interpretation of the results from our study. FIGO stage information was confirmed by clinical review and enabled the assessment of not only the cancer preventive, but also the downstaging potential of screening, although with limited power.

Although not all age-eligible women were included in the study population due to incomplete registration in some counties, there was evidence of only small differences in the baseline characteristics of women who could and could not be included, and our sensitivity analyses restricting to later birth cohorts did not present inconsistent results. Thus, the results appear generalizable to the whole population. As only some counties in the nation have implemented organized screening for women aged 61–65, women who were screened in counties without organized screening may differ systematically from those in counties offering organized screening for these ages. Therefore, we performed a sensitivity analysis restricting the investigation of screening effects to counties where a large proportion of women were screened at age 61–65, and determined that the results were stable (Table D in S2 Text). We further adjusted for educational level, since this may correlate with health consciousness, screening attendance, and other health-related behaviors that may have spuriously biased the association in favor of screening. In sensitivity analyses, we also adjusted for lifetime diagnosis of COPD as a proxy for smoking status, as well as parity, with unaltered point estimates (Table D in S2 Text). Thus, none of our investigations showed evidence of major confounding. We hold it likely that the consistent stratification on screening history already absorbed a large amount of potential confounding, as participants with the same screening history tended to have similar healthcare behavior. There could still be residual confounding effects from factors such as sexual behavior. If high-risk sexual behavior is associated with low screening participation in women aged 61–65, an overestimation of the benefit of screening may occur. However, data from a survey in young Swedish women [29] revealed that cervical screening attendance is only marginally associated with sexual behavior. We hold it unlikely that this factor substantially confounded the observed results.

### Other considerations

The potentially improved protection and greater efficiency derived from HPV testing over cytology in older women should be considered in this context, due to the evidence that (1) the sensitivity of HPV primary testing for detecting CIN2+ (cervical intraepithelial neoplasia grade 2 or worse) is generally higher than that of cytology, and the specificity is close to that of cytology for women age 50 and above [30] and (2) HPV infection is less likely to be newly acquired at these ages [31,32], and a new infection is cleared much faster [32] and is less likely to progress to CIN2+ disease [31]. A normal HPV test result therefore may provide longer protection than a normal Pap test result. A pooled analysis of 4 randomized trials in Europe showed that primary HPV testing above the age of 50 may prevent 32% more future cervical cancer cases than cytology [33]. To optimize a screening strategy for older women based on HPV testing, the following items need to be investigated: (1) whether HPV testing after age 60 further reduces the cancer risk for women screened with cytological normal results in their 50s and (2) the subsequent risk profile and magnitude of benefit from HPV testing after age 60 in women screened with HPV-negative results in their 50s. Although historical screening data are limited since they do not include HPV-based screening, we present here a structured model for future studies that can be applied to further investigate at which age and with which criteria to discontinue HPV-based screening.

It should be noted that our study addressed only 1 age span, 61–65 years, due to limited data from screening of women of older ages. Uncertainty therefore remains about the effectiveness of screening at older ages than 61–65. A modeling study has suggested that continued screening after age 65 provides little additional benefit for incidence and mortality among women screened with cytology every 3 years before age 65 [34]. Based on this, the US guidelines recommend discontinuing screening for women older than 65 if they have been adequately screened with no abnormality until that age [35]. In future studies, it would be of great interest to investigate whether screening at even older ages would benefit women previously unscreened or showing abnormalities.

A previous study from Sweden showed that women who have had a cervical cancer in situ diagnosis sometime in their lifetime are at high risk of developing cervical cancer at older ages even if it was treated [36]. This finding suggests that besides women’s screening history in their 50s, diagnosis of cervical cancer in situ at earlier ages also needs to be considered when designing the cervical screening strategy for older aged women.

### Conclusions and policy implications

In this study, we showed that cervical screening with cytology at age 61–65 was associated with a statistically significant cervical cancer risk reduction for women who were unscreened, or screened with abnormalities, in their 50s. It should thus be reasonable to conclude that these women should continue to be screened after age 60. In women who were screened with normal results in their 50s, the risk of future cancer was not sizeable, and the risk reduction afforded by continued screening appeared very limited. However, continued cervical screening at age 61–65 was still associated with downstaging of residual cancer cases. The choice of further screening strategy for this group of women should therefore be based on the availability of resources in each context.

Our study informs the current debate regarding at which age and with which criteria to discontinue cervical screening, and presents a structured model for future research necessary to investigate the impact of HPV-based screening. Our results should provide guidance for cervical screening programs on how to reach a satisfactory cost–benefit balance when screening older women, especially under the likely scenario that an increasing proportion of women at older ages will have been adequately screened in their 50s.

## Supporting information

S1 STROBE Checklist (DOCX)

S1 Fig Time of initiating cervical screening records by counties/hospitals. (PNG)

S2 Fig Distribution of the time between Pap test and cancer diagnosis for distinguishing diagnostic workup Pap test from screening Pap test, in women diagnosed with invasive cervical cancer at age 61–65 years in 2002–2011. Mode of detection of cervical cancer (i.e., screen-detected or symptomatic) was retrieved from medical charts. It was then linked with their screening record prior to cancer diagnosis. We plotted the time between cancer diagnosis and the first abnormal smear in the year prior to cancer diagnosis, by mode of detection. Around 85% of the symptomatic cancer cases had their first abnormal smear within 50 days prior to their cancer diagnosis, and around 75% of screen-detected cancer cases had their first abnormal smear more than 50 days prior to their cancer diagnosis. Overall, considering the proportion of screen-detected and symptomatic cancer cases, a 50-day cutoff gave the highest accuracy rate of correct classification for this age group. Sensitivity analyses using cutoffs of 30 and 40 days were also performed. Results are shown in Table D in S2 Text. The odds ratios were not significantly different from the main analysis.
(PNG)

S1 Text Analysis protocol. (DOCX)

S2 Text Supporting tables. (DOCX)

## Abbreviations

COPD: chronic obstructive pulmonary disease
HR: hazard ratio
NCR: Swedish National Cancer Registry
NKCx: Swedish National Cervical Screening Registry
TPR: Total Population Register
