# Supplementary figures and images for: Effectiveness of cervical screening after age 60 years according to screening history: Nationwide cohort study in Sweden
Source: PLoS Med. 2017 Oct 24;14(10):e1002414. doi: 10.1371/journal.pmed.1002414 (PMC5655486; doi:10.1371/journal.pmed.1002414)

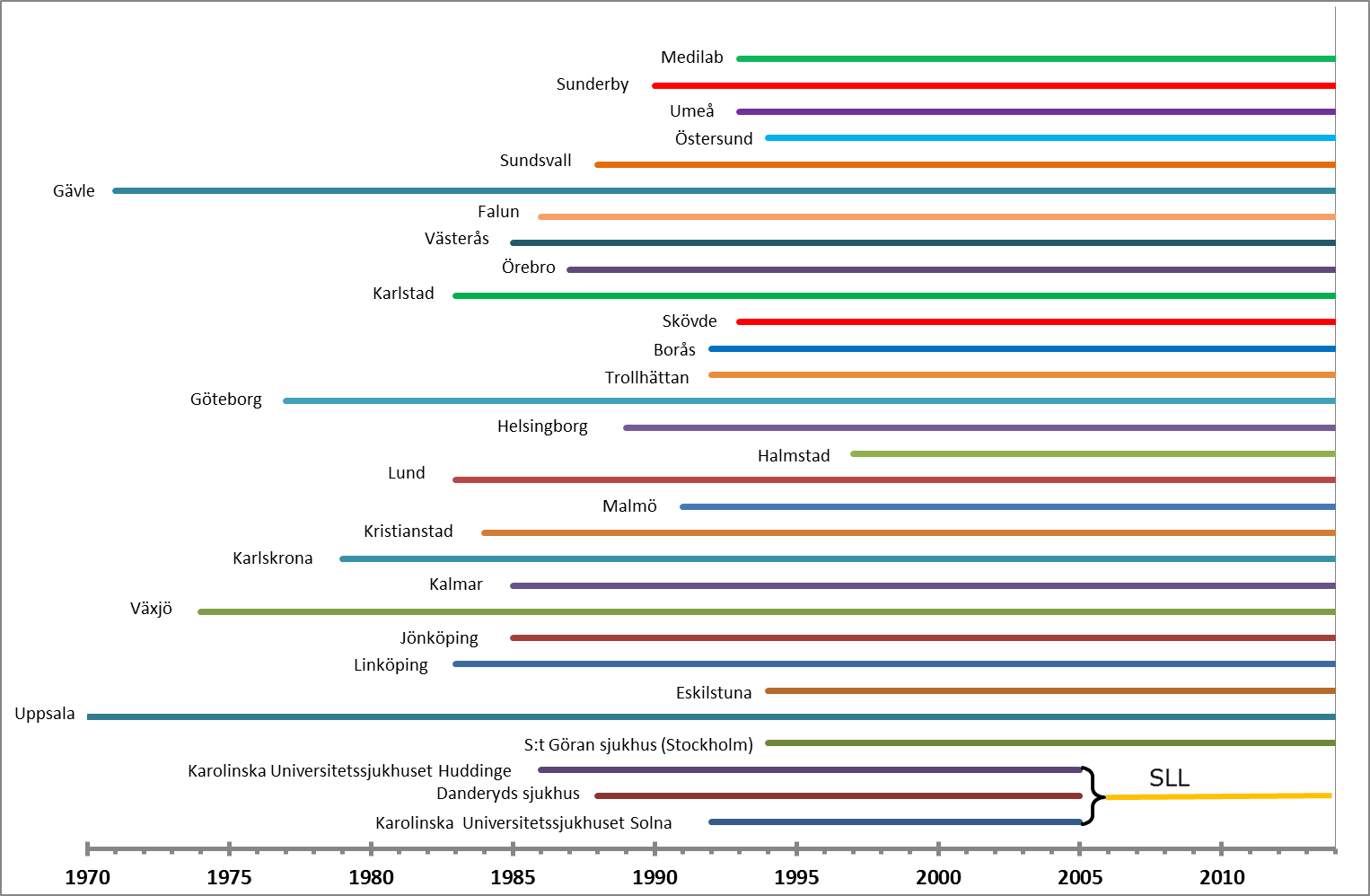

Supplement: S1 Fig — (PNG) [file pmed.1002414.s002.png]

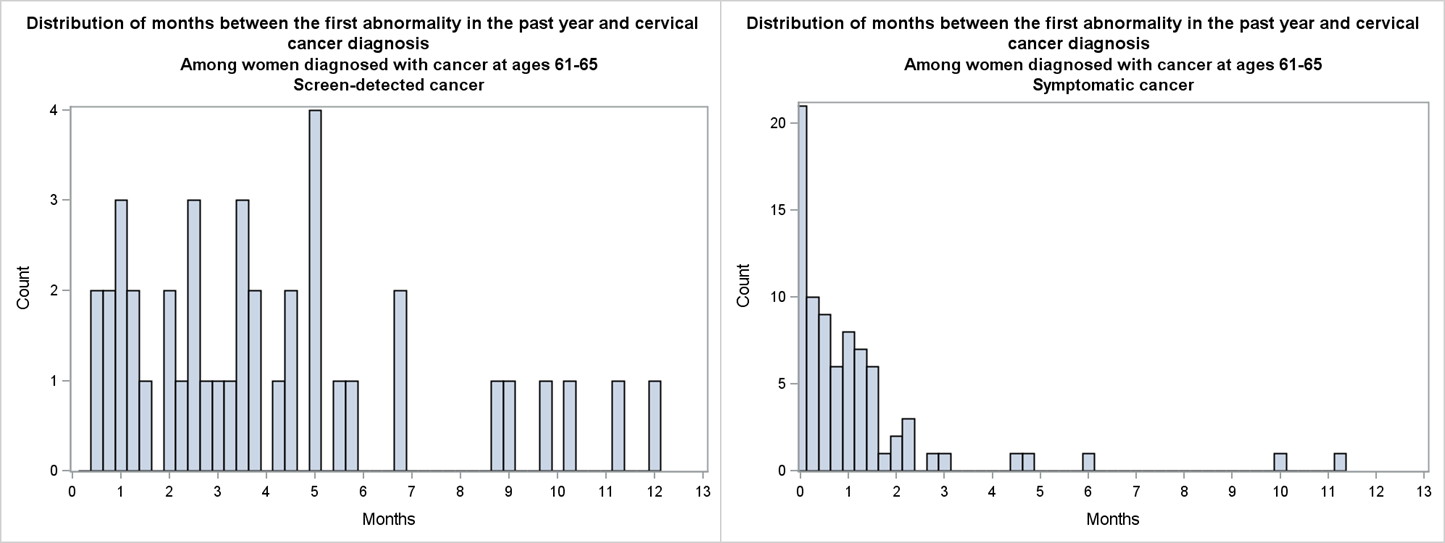

Supplement: S2 Fig — Mode of detection of cervical cancer (i.e., screen-detected or symptomatic) was retrieved from medical charts. It was then linked with their screening record prior to cancer diagnosis. We plotted the time between cancer diagnosis and the first abnormal smear in the year prior to cancer diagnosis, by mode of detection. Around 85% of the symptomatic cancer cases had their first abnormal smear within 50 days prior to their cancer diagnosis, and around 75% of screen-detected cancer cases had their first abnormal smear more than 50 days prior to their cancer diagnosis. Overall, considering the proportion of screen-detected and symptomatic cancer cases, a 50-day cutoff gave the highest accuracy rate of correct classification for this age group. Sensitivity analyses using cutoffs of 30 and 40 days were also performed. Results are shown in Table D in S2 Text. The odds ratios were not significantly different from the main analysis. (PNG) [file pmed.1002414.s003.png]
