# Supplementary material for: Effectiveness of cervical screening after age 60 years according to screening history: Nationwide cohort study in Sweden
Source: PLoS Med. 2017 Oct 24;14(10):e1002414. doi: 10.1371/journal.pmed.1002414 (PMC5655486; doi:10.1371/journal.pmed.1002414)
Supplement: S1 Text — (DOCX) [file pmed.1002414.s004.docx]

**Analysis plan - First version**

**Created: 2013-07-01**

# OBJECTIVE AND HYPOTHESES

**Aim:** to evaluate the effectiveness of cervical cancer screening at ages 51-65 for preventing invasive cervical cancer after 65 years of age; to identify women at high risk of developing cervical cancer after age 65, in relation to their screening history between the ages of 51-60.

**Hypotheses:**

1. Having two normal smears at ages 51-60 is effective for preventing cancer after 65;
2. Partial or complete absence of screening during ages 51-60 confer higher risk of cancer after 65;
3. Having any abnormal smear during ages 51-60 confer higher risk of cancer after 65;
4. One additional normal smear during ages 61-65 can offer better protection;
5. One additional normal smear during ages 61-65 can make up one or two absence in 50-60 years of age.

# STUDY POPULATION

Cervical cancer cases at ages 66-80, from 2002-2010

30 controls for each case, randomly select from population, matched on birth-year and county of residence.

# MEASUREMENTS AND VARIABLES

## Outcome variables

Case = case indicator of cervical cancer.

1=case, 0=control.

## Exposure covariates

Original variables:

Cat1 = indicator for smear in age 51-55.

0 = no smear

1 = only normal smear(s)

3 = at least one abnormal smear, the most sever abnormality is high-grade.

2 = at least one abnormal smear, the most sever abnormality is low-grade.

Cat2 = indicator for smear in age 56-60.

0 = no smear

1 = only normal smear(s)

3 = at least one abnormal smear, the most sever abnormality is high-grade.

2 = at least one abnormal smear, the most sever abnormality is low-grade.

Cat3 = indicator for smear in age 61-65.

0 = no smear

1 = only normal smear(s)

3 = at least one abnormal smear, the most sever abnormality is high-grade.

2 = at least one abnormal smear, the most sever abnormality is low-grade.

1. Screening history at ages 51-60:
2. Adequately screened with negative results: cat1=0 and cat2=0 and the first and the last smear during 51-60 should be at least one year apart
3. Inadequately screened with negative results: cat1=0 or cat2=0
4. Unscreened: cat1=0 and cat2=0
5. Low-grade abnormality: [cat1=2 and (cat2=0 or 1 or 2)] or [(cat1=0 or 1 or 2) and cat2=2]
6. High-grade abnormality: [cat1=3 and (cat2=0 or 1 or 2 or 3)] or [(cat1=0 or 1 or 2 or 3) and cat2=3]
7. Screening history at ages 61-65
8. Screened with normal result(s): cat3=1
9. Unscreened: cat3=0
10. Screened with abnormal results: cat3=2 or 3

# STATISTICAL ANALYSES

## Statistical methods:

Conditional logistic regression models

## Comparison plan for each hypothesis:

| **51-60** | **61-65** | **Comp.**  **Hypothesis**  **1** | **Comp. Hypothesis 2** | **Comp. Hypothesis 3** | **Comp. Hypothesis 4** | **Comp. Hypothesis 5** |
| --- | --- | --- | --- | --- | --- | --- |
| iii | ii | **Ref.** | **×** |  |  | **Ref.** |
| i | ii | **×** | **Ref.** | **Ref.** | **Ref.** |  |
| ii | ii |  | **×** |  |  | **Ref.** |
| iv or v | ii |  |  | **×** |  |  |
| iii | i |  |  |  |  | **×** |
| i | i |  |  |  | **×** |  |
| ii | i |  |  |  |  | **×** |
| iv or v | i |  |  |  |  |  |

**Analysis plan – Updated version**

**Created: 2015-06-15**

# OBJECTIVE AND HYPOTHESES

**Aim:** To investigate the effectiveness and the impact of cervical screening at ages 61-65 stratifying by screening history at ages 51-60.

**Hypotheses:**

1. Women screened at ages 61-65 had lower risk of cervical cancer up to age 80 years compared to those unscreened, among women with all screening history categories at ages 51-60;
2. Women screened at ages 61-65 had lower risk of advanced cervical cancer up to age 80 years compared to those unscreened, among all screening history categories at ages 51-60;

# STUDY POPULATION

## Inclusion criteria

- Swedish reside women born between **191901** and **194512** (at ages 51 or younger when 197001-the initiation of the record in The Swedish National Cervical Screening Registry (NKCx); and older than age 65 at the end of cancer diagnoses record 20111231).
- Screening records available since age 51 (each county has a year that start to have electronic record of smears (see graph in NKCx annual report), women living in the county should be at age 51 or younger at that year)

## Exclusion criteria

- Invalid or missing PID;
- Not alive or not live in Sweden since age 51;
- Invasive CxCa before age 61;
- Total hysterectomy before age 61;
- Emigrated before age 61;
- Dead before age 61;

# MEASUREMENTS AND VARIABLES

## Outcome

Invasive cervical cancer occurrence at ages 61-80, and FIGO stage (2002-2011 cases from medical charts)

Censoring criteria: emigrated; dead; total hysterectomized

## Exposure covariates

### The main exposure:

Screening status 61-65 (diagnostic smears were removed: smears within 50 days prior to cervical cancer diagnosis): identify the first smear at ages 61-65; contribute to unscreened group before the first smear and then contribute to screened group since the first smear

### The stratification exposure

Screening history at ages 51-60

Original variables:

Cat1 = indicator for smear in age 51-55.

0 = no smear

1 = only normal smear(s)

3 = at least one abnormal smear, the most sever abnormality is high-grade.

2 = at least one abnormal smear, the most sever abnormality is low-grade.

Cat2 = indicator for smear in age 56-60.

0 = no smear

1 = only normal smear(s)

3 = at least one abnormal smear, the most sever abnormality is high-grade.

2 = at least one abnormal smear, the most sever abnormality is low-grade.

1. Adequately screened with negative results: cat1=0 and cat2=0 and the first and the last smear during 51-60 should be at least one year apart
2. Inadequately screened with negative results: cat1=0 or cat2=0
3. Unscreened: cat1=0 and cat2=0
4. Low-grade abnormality: [cat1=2 and (cat2=0 or 1 or 2)] or [(cat1=0 or 1 or 2) and cat2=2]
5. High-grade abnormality: [cat1=3 and (cat2=0 or 1 or 2 or 3)] or [(cat1=0 or 1 or 2 or 3) and cat2=3]

## Mandatory covariates, known confounders

Education level:

1) low: less than high school;

2) middle: high school;

3) high: university studies and above

Calendar year of birth:

1919-1925

1926-1930

1931-1935

1936-1940

1941-1945

## Additional covariates, potential confounders

COPD(Life-time): proxy for smoking status: from patient register

In-patient register 1964-2011, Out-patient register 2001-2011. Diagnoses were almost half in inpatient and half in outpatient register, so incomplete for the whole population.

Parity: number of children from Multi-generation register, as a continuous variable

Register only started since birth cohort of 1932.

# STATISTICAL ANALYSES

## Cumulative incidence:

Cumulative incidence curves of cervical cancer at ages 61-80: time-dependent covariate of screening status 61-65, stratified by screening history at ages 51-60 (use stcompete command in stata, considering death and hysterectomy as competing events)

## Relative incidence

Hazard ratios from cox regression, comparing screened to unscreened at ages 61-65, stratified by screening history at ages 51-60. Present crude HR and adjusted HR (adjust for education and birth cohort)

## Stage distribution of cervical cancer

Require linkage with the new audit data with cervical cancer cases during year 2002-2011

Present distribution of cancer stage (IA, IB and II+), and calculate proportional odds ratio and p-values comparing screened to unscreened at ages 61-65 , stratified by screening history at ages 51-60.

# SENSITIVITY/VALIDATION ANALYSES

## Potential difference between women included in the study population and not included due to data availability issue:

Present distribution of education level, and calculated odds ratios and p-values adjusted for birth-cohort

## Validate the 50-day time-frame to defining diagnostic pap smears at aged 61-65

Use the audit database with cases in 2002-2011 because it has mode of detection (screen-detected or symptomatic cancer) from the medical chart. Then link with screening data, distribute time between the first abnormal smear in the past year and cancer diagnosis. Compare the time between screen-detected and symptomatic cancer.

## Sensitivity analyses adjusting for COPD and parity

In the Cox regression, based on the adjusted model, further adjust for COPD for the whole population, and parity for birth cohort 1932-1945

## Sensitivity analyses of time-frame 30 days and 40 days for defining diagnostic smear

Present adjusted HRs from cox regression, remove smears within 30 or 40 days before cancer diagnosis at ages 61-65.

## Sensitivity analyses within counties that had more than 40% of women being screened at ages 61-65

To minimize the potential bias if have very few women being screened in a county, because women being screened can have more extreme characteristics.

Identify those counties and the starting years in the NKCx screening data.

Present adjusted HRs from cox regression within those counties.

## Sensitivity analyses stratifying by birth cohort

To assess potential calendar period effect, and potential difference due to completion of the data, i.e. proportion of women having screening record since age 51.

Present adjusted HRs from cox regression in birth cohorts 1919-1945, 1926-1945, 1931-1945 and 1936-1945.

## Sensitivity analyses comparing screening effect at ages 56-60 to that at ages 61-65

Examine the effect of cervical screening at ages 56-60: in women screened with normal results at ages 51-55, assess the hazard ratio of cervical cancer at ages 56-80 comparing screened to unscreened at ages 56-60, from cox regression. If women screened at ages 61-65, censor the follow-up at the first smear at ages 61-65.

**REASONS FOR REVISING ANALYSIS PLAN:**

1. In the case-control study, cervical cancer cases from year 2001-2010 does not provide sufficient power, especially for analyses in the group of women with abnormal screening history in their 50s, which is a very small group.
2. The case-control database is not linked with information of education, migration, death and other related factors, which affect the validity and accuracy of the study.
3. A similar study using case-control design has been published (Castanon, et, al. PLOS Medicine, 2014).
4. The research question is refined to accommodate the more important evidence demand regarding time and criteria to discontinue cervical screening, for which a cohort design is more appropriate and higher in level of evidence.
