# Supplementary material for: Effectiveness of cervical screening after age 60 years according to screening history: Nationwide cohort study in Sweden
Source: PLoS Med. 2017 Oct 24;14(10):e1002414. doi: 10.1371/journal.pmed.1002414 (PMC5655486; doi:10.1371/journal.pmed.1002414)
Supplement: S2 Text — (DOCX) [file pmed.1002414.s005.docx]

# S2 Text. Supporting tables

## Table A. SNOMED codes for cytological diagnoses in the study, defined by the Swedish Association for Clinical Cytology

| Cytological diagnosis | SNOMED code |
| --- | --- |
| Normal cytology | M00110 |
| Low-grade abnormalities |  |
| Atypical squamous cells of undetermined significance | M69710 |
| Mild squamous dysplasia | M74006 |
| High-grade abnormalities |  |
| Suspected high-grade squamous dysplasia | M69719 |
| Moderate squamous dysplasia | M74007 |
| Severe squamous dysplasia | M80702 |
| Atypical glandular cells | M69720 |
| Atypical cells of uncertain origin | M69700 |
| Squamous cell cancer | M80703 |
| Adenocarcinoma/Adenocarcinoma in situ | M81403 |

## Table B. Level of education of women included and excluded due to lack of screening record in this study

|  | Education level | | | |
| --- | --- | --- | --- | --- |
|  | Low | Middle | High | Missing |
| In the study population (row %) | 256743 (39.9) | 244723 (38.0) | 132754 (20.6) | 9303 (1.5) |
| Not in the study population (row %) | 390187 (55.3) | 192700 (27.3) | 71509 (10.1) | 51820 (7.3) |
| Adjusted Odds Ratio (95% CI) ^a^ | REF. | 1.09 (1.08-1.10) | 1.11 (1.10-1.13) | 1.35 (1.32-1.39) |

a. In relation to the lowest education level, estimated from a logistic regression model adjusted for birth cohort.

Although the frequency table shows an obvious difference in level of education between women in and out of the study population, it is highly likely to be confounded by birth cohort, because younger birth cohorts had more women included in the study population, and younger birth cohorts were also better educated. We therefore conducted the logistic regression to assess the association adjusting for birth cohorts.

According to the odds ratio, women in the study population were more likely to be at middle or high education level compared to women not in, after adjusting for birth cohort. Although the differences were statistically significant (95%CI not includes 1), the odds ratio itself was close to 1, which means the association was not strong. The association for the group with missing data on education was stronger, but only applied to a fraction of the population.

## Table C. Sub-distribution hazard ratio of cervical cancer from age 61 to 80 years comparing women screened to unscreened at age 61-65 years, by screening history at age 51-60 years, based on a Fine-Gray model for the cumulative incidence function

| Screen history at age 51-60 years | Unadjusted model | | Adjusted for birth-cohort and education | |
| --- | --- | --- | --- | --- |
|  | SHR (95%CI)^a^ | P-value for PH test^b^ | SHR (95%CI) ^a^ | P-value for PH test^b^ |
| Adequately screened, normal | 0.95(0.72-1.25) | 0.11 | 0.91(0.69-1.20) | 0.19 |
| Inadequately screened, normal | 0.90(0.61-1.32) | 0.43 | 0.84(0.57-1.25) | 0.59 |
| Unscreened | 0.39(0.23-0.67) | 0.56 | 0.42(0.25-0.73) | 0.52 |
| Low-grade abnormality | 0.42(0.24-0.73) | 0.62 | 0.44(0.25-0.78) | 0.65 |
| High-grade abnormality | 0.61(0.37-1.01) | 0.31 | 0.61(0.36-1.02) | 0.27 |

1. Sub-distribution hazard ratio and 95% confidence interval, comparing women screened to unscreened at age 61-65
2. P-value for proportional hazard test

## Table D. Hazard ratio and confidence interval of cervical cancer from age 61 to 80 years, comparing women screened to unscreened at age 61-65 years, by screening history at age 51-60 years from sensitivity analyses

| Screen history at age 51-60 years | Original analysis^a^ | Further adjusted for COPD ^b^ | Further adjusted for parity ^c^ in birth cohorts 1932-1945 | Within counties with >40% women screened at age 61-65 | Time-frame^d^ 40 days between Pap test and cancer diagnosis | Time-frame^d^ 30 days between Pap test and cancer diagnosis |
| --- | --- | --- | --- | --- | --- | --- |
| Adequately screened, normal | 0.90(0.69-1.17) | 0.90(0.69-1.17) | 0.93 (0.68-1.26) | 1.07(0.77-1.47) | 0.93(0.71-1.21) | 1.02(0.78-1.32) |
| Inadequately screened, normal | 0.82(0.56-1.22) | 0.83(0.56-1.22) | 0.80 (0.50-1.28) | 0.82(0.51-1.34) | 0.96(0.66-1.40) | 1.03(0.71-1.49) |
| Unscreened | 0.42(0.24-0.72) | 0.42(0.24-0.72) | 0.57 (0.30-1.10) | 0.42(0.22-0.80) | 0.45(0.27-0.76) | 0.55(0.34-0.89) |
| Low-grade abnormality | 0.43(0.25-0.74) | 0.43(0.25-0.74) | 0.51 (0.24-1.07) | 0.57(0.29-1.10) | 0.43(0.25-0.74) | 0.46(0.27-0.80) |
| High-grade abnormality | 0.59(0.36-0.96) | 0.59(0.36-0.96) | 0.43 (0.24-0.79) | 0.51(0.29-0.91) | 0.59(0.36-0.96) | 0.67(0.41-1.10) |

1. Adjusted for birth-cohort and education
2. Chronic obstructive pulmonary disease, retrieved from the Patient Register. The register is a combination of inpatient register (initiated in 1964) and outpatient register (initiated in 2005). As COPD is frequently diagnosed in out-patient practice, note that data on COPD are not complete in the cohort before 2005.
3. Parity is retrieved from the Swedish Multi-Generation Register which records mother-child relationship of the whole country from birth cohort 1932 onwards. It is treated as a continuous variable. The significance of the effect of screening at ages 61-65 among women unscreened or having abnormalities in their 50s is loss, due to the limit size of study population when restricting to later birth cohorts. But all point estimates were almost consistence with the main analysis.
4. Time-frame for distinguishing screening test and cancer diagnostic test.

## Table E. Adjusted hazard ratio and confidence interval of cervical cancer from age 61-80 in different birth cohorts

| Screen history at age 51-60 years | Adjusted HR^a^ comparing women screened to unscreened at age 61-65 | | | |
| --- | --- | --- | --- | --- |
|  | Birth cohort 1919-1945 | Birth cohort 1926-1945 | Birth cohort 1931-1945 | Birth cohort 1936-1945  (the longest follow-up until age 75) |
| Adequately screened, normal | 0.90(0.69-1.17) | 0.84(0.63-1.10) | 0.89(0.66-1.21) | 1.18(0.84-1.65) |
| Inadequately screened, normal | 0.82(0.56-1.22) | 0.80(0.53-1.22) | 0.74(0.47-1.18) | 0.86(0.47-1.56) |
| Unscreened | 0.42(0.24-0.72) | 0.40(0.23-0.72) | 0.56(0.30-1.04) | 1.10(0.57-2.11)^b^ |
| Low-grade abnormality | 0.43(0.25-0.74) | 0.45(0.25-0.81) | 0.47(0.24-0.94) | 0.57(0.24-1.37) |
| High-grade abnormality | 0.59(0.36-0.96) | 0.54(0.32-0.90) | 0.47(0.26-0.83) | 0.66(0.32-1.34) |

1. Hazard ratios adjusting for birth cohort and education
2. This result is inconclusive, because much less women in the later birth cohorts were unscreened in their 50s compared to earlier birth cohorts, and those unscreened in 50s are also likely to be unscreened after 60. Thus very few women (3779, even less than the previous abnormal groups) were unscreened at 51-60 and screened at 61-65, and only 10 cervical cancer cases in this category. This makes the power very limited, and the result unstable compared to the other birth cohorts.

## Table F. Hazard ratio of cervical cancer at age 56-80 years comparing women screened and unscreened at age 56-60 years, among women screened normal at ages 51-55

|  | No. of women | No. of cervical cancer cases | Person-years per 100,000 | Crude incidence rate per 100,000 person-years | HR (95%CI) ^a^ | HR adjusted for education and birth cohort |
| --- | --- | --- | --- | --- | --- | --- |
| Screening status at age 56-60 years |  |  |  |  |  |  |
| Unscreened | 432012 | 186 | 18.0 | 10.3 | Ref. | Ref. |
| Screened | 358279 | 363 | 47.3 | 7.7 | 0.66 (0.53-0.81) | 0.69 (0.55-0.86) |

1. Hazard ratio and 95% confidence interval from interaction model with time-varying exposure of screening status at ages 56-60 and 61-65

In this supplementary analysis, we estimated the effect of screening at age 56-60 years among those screened with normal results at age 51-55. During the follow-up time, only those unscreened after age 60 contributed to the risk-time up to age 80 (i.e. women screened after age 60 were censored when the first Pap test was taken). This is to rule out the influence of a further screening test.

The effect of screening at age 56-60 years is larger (HR=0.69, 95%CI=0.55-0.86) than the effect of screening at age 61-65 years in women previously screened with normal result in their 50s (HR=0.90, 95%CI=0.69-1.17 in Table 3).
